# GO-2D: identifying 2-dimensional cellular-localized functional modules in Gene Ontology

**DOI:** 10.1186/1471-2164-8-30

**Published:** 2007-01-24

**Authors:** Jing Zhu, Jing Wang, Zheng Guo, Min Zhang, Da Yang, Yanhui Li, Dong Wang, Guohua Xiao

**Affiliations:** 1 Department of Bioinformatics, Harbin Medical University, Harbin 150086, China; 2 Department of Pharmacology and Bio-pharmaceutical Key Laboratory of Heilongjiang Province and State, Harbin Medical University, Harbin 150086, China

## Abstract

**Background:**

Rapid progress in high-throughput biotechnologies (e.g. microarrays) and exponential accumulation of gene functional knowledge make it promising for systematic understanding of complex human diseases at functional modules level. Based on Gene Ontology, a large number of automatic tools have been developed for the functional analysis and biological interpretation of the high-throughput microarray data.

**Results:**

Different from the existing tools such as Onto-Express and FatiGO, we develop a tool named GO-2D for identifying 2-dimensional functional modules based on combined GO categories. For example, it refines biological process categories by sorting their genes into different cellular component categories, and then extracts those combined categories enriched with the interesting genes (e.g., the differentially expressed genes) for identifying the cellular-localized functional modules. Applications of GO-2D to the analyses of two human cancer datasets show that very specific disease-relevant processes can be identified by using cellular location information.

**Conclusion:**

For studying complex human diseases, GO-2D can extract functionally compact and detailed modules such as the cellular-localized ones, characterizing disease-relevant modules in terms of both biological processes and cellular locations. The application results clearly demonstrate that 2-dimensional approach complementary to current 1-dimensional approach is powerful for finding modules highly relevant to diseases.

## Background

It is widely accepted that functionally related genes tend to express and perform their highly concerted cellular functions in some isolated and interactive modular fashions [1,2]. Global gene expression data have provided an opportunity for understanding the transcriptional modularity characterizing complex diseases [3-6]. For example, Mootha et al. [6] showed that the coordinate disease-associated changes of a set of functionally related genes could be identified even when the expression of individual genes changes modestly. Segal et al. [3] defined 'modules' as gene sets that are conditionally activated or repressed across a wide variety of cancer types, and identified some modules deregulated in cancer. Our recent study demonstrated that based on functional modules, i.e., GO categories enriched with differentially expressed genes (DEGs), cancer types can be precisely and robustly classified by supervised classification analysis [5] or discovered by clustering analysis [7].

For high-throughput microarray data analysis, translating lists of interesting genes (e.g., DEGs) into functional modules for understanding the biological phenomena has become an important routine task. Based on Gene Ontology, a large number of tools such as Onto-Express [8], FatiGO [9], GoMiner [10] and GOstat [11] have been developed for this purpose. However, most existing approaches interpret the interesting genes using categories from three ontologies "biological process" (BP), "molecular function" (MF) and "cellular component" (CC) separately, which may be inefficient for mapping some specific modular activities in cells. For example, a GO BP category usually encompasses the genes involved in distinct processes occurring in different cellular compartments [12], and the genes even within a same process may show a clear expression distinction with respect to their cellular localizations [13]. Therefore, in this paper, by combining categories from BP, CC, and MF, we propose GO-2D as a tool for finding 2-dimensional functional modules (e.g., the cellular-localized modules) for studying complex human diseases.

We use two cancer datasets for numerical analysis, and the results show that with the same FDR (false discovery rate) criteria, many specific processes relevant to diseases cannot be found until additionally cellular location information is used. The results clearly demonstrate the insufficiency of current 1-dimensional approaches and highlight the importance of using 2-dimensional modules for disease analysis.

## Implementation

GO-2D has been implemented in JAVA and interconnected to a relational database system by using MS-Access 2000 for Windows version and SQLite for Linux version.

### Database

In GO-2D, associations of gene IDs from different organisms (including *Homo sapiens*, *Drosophila melanogaster*, *Caenorhabditis elegans*, *and Saccharomyces cerevisiae*) to GO terms are based on the databases Gene, SGD, FlyBase, and WormBase. Tables relating GO terms with gene IDs can be found in the NCBI web page [14] and GO Consortium web page [15]. The Unigene build #190 is used in GO-2D.

### Analysis and visualization

**Figure 1:**
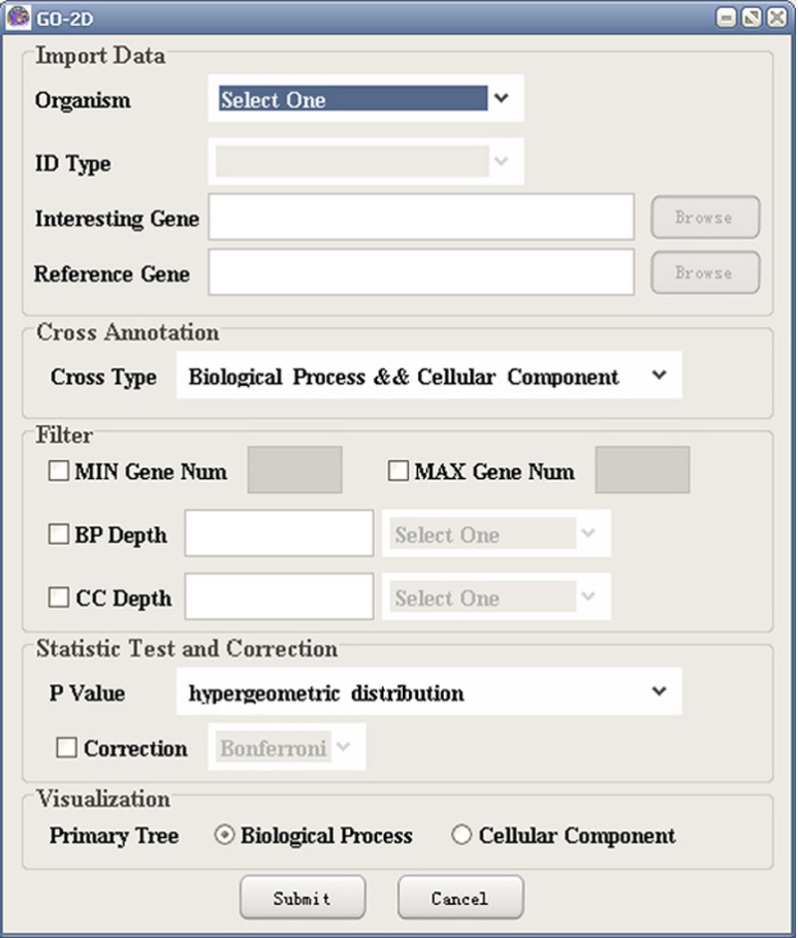
A snapshot of GO-2D: the main user interface.

**Figure 2:**
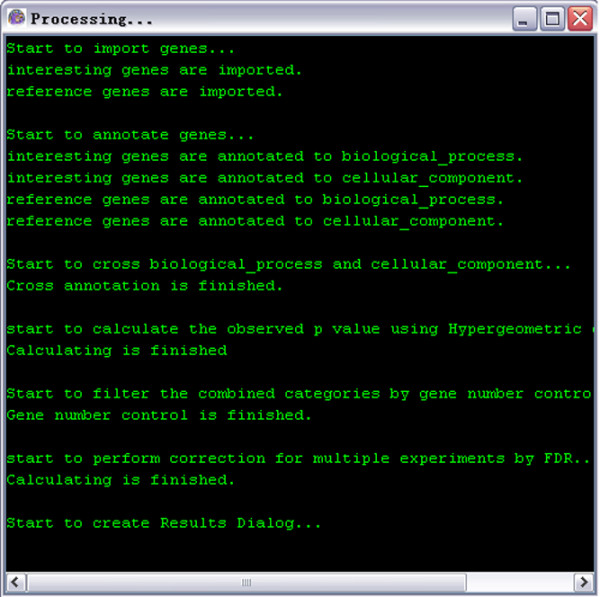
A snapshot of GO-2D: the processing page.

**Figure 3:**
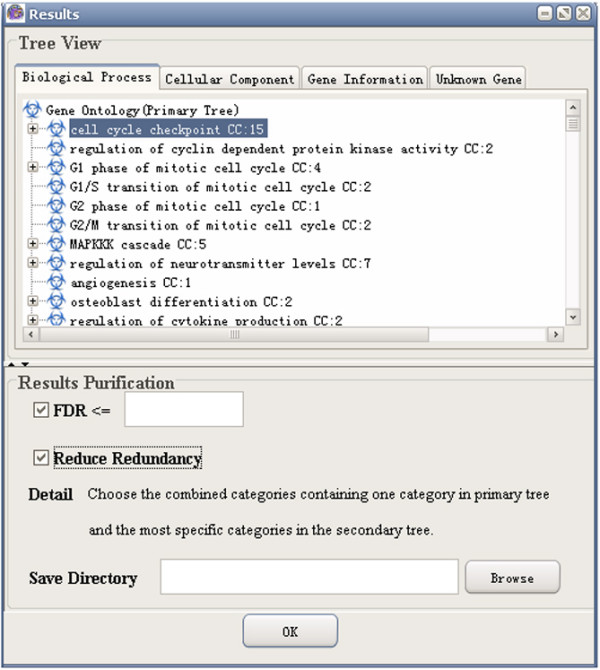
A snapshot of GO-2D: the results page.

Data analysis is made flexible by subdividing the procedure into sequential steps:

(1) Import data: GO-2D starts by reading the input files containing reference and interesting gene lists (see Figure 1). It queries the genes by using Entrez Gene and Unigene for human and organism specific IDs in GO for the other three species (*Drosophila melanogaster*, *Caenorhabditis elegans*, *and Saccharomyces cerevisiae*).

(2) Cross annotation: GO-2D refines a BP category by sorting its genes into different CCs to form combined categories for finding cellular-localized modules enriched with the interesting genes (see Figure 2). It also provides the other 2-dimensional combinations of categories from the three ontologies (BP, MF, and CC).

(3) Filter data: GO-2D provides options for finding general or specific combined categories by determining their sizes (the minimum/maximum numbers of included genes) and/or depths in GO.

(4) Statistic test: GO-2D calculates the probability of a combined category having the annotated number of interesting genes by random chance, based on hypergeometric or binomial statistical model [8], which is named "the observed *p *value".

(5) Multiple tests correction: GO-2D offers Bonferroni correction and FDR control [16] for multiple statistical tests, the results are shown as "the corrected *p *value". When a total of *n *combined categories are tested, for the Bonferroni correction, the corrected *p *value is *pn*, while *p *is the observed *p *value. For the FDR control, let *p *(*k*) denote the *k*-th smallest observed *p *value in a total of *n *combined categories, then the FDR *f*_*k *_for hypothesis *k *is bounded by *np*(*k*)/*k *≤ *f*_*k*_. If an FDR of *f *is required for the entire experiment, all hypotheses that satisfy *p*(*k*) ≤ *fk*/*n *are declared as significant. The corrected *p *value for the *k*-th smallest observed *p *value is *np*(*k*)/*k*. GO-2D can also output all the observed *p *values, which can be used for other complicated multiple tests correction by many other existing tools such as the program for Storey's *Q *value [17].

(6) Results: GO-2D allows users to save the results for detailed examination of the identified modules. The tabular results collect the following information of a combined category: GO IDs, names and depths of categories (e.g. both BP and CC), numbers of genes and interesting genes annotated in it, the observed p values, and the corrected p values for multiple tests of the combined categories.

(7) Results visualization: GO-2D also provides tree view to visualize the 2-dimensional modules (e.g. BP and CC). GO-2D firstly displays the primary categories (e.g. BP, user defined) in the primary tree, and then in the secondary tree, shows the sub-hierarchical structure of the secondary categories (e.g. CC) within each primary category (see Figure 3). The user can select either BP or CC as the "primary tree" for visualization. The selection has no effect on the calculation of over-represented categories.

**Figure 4:**
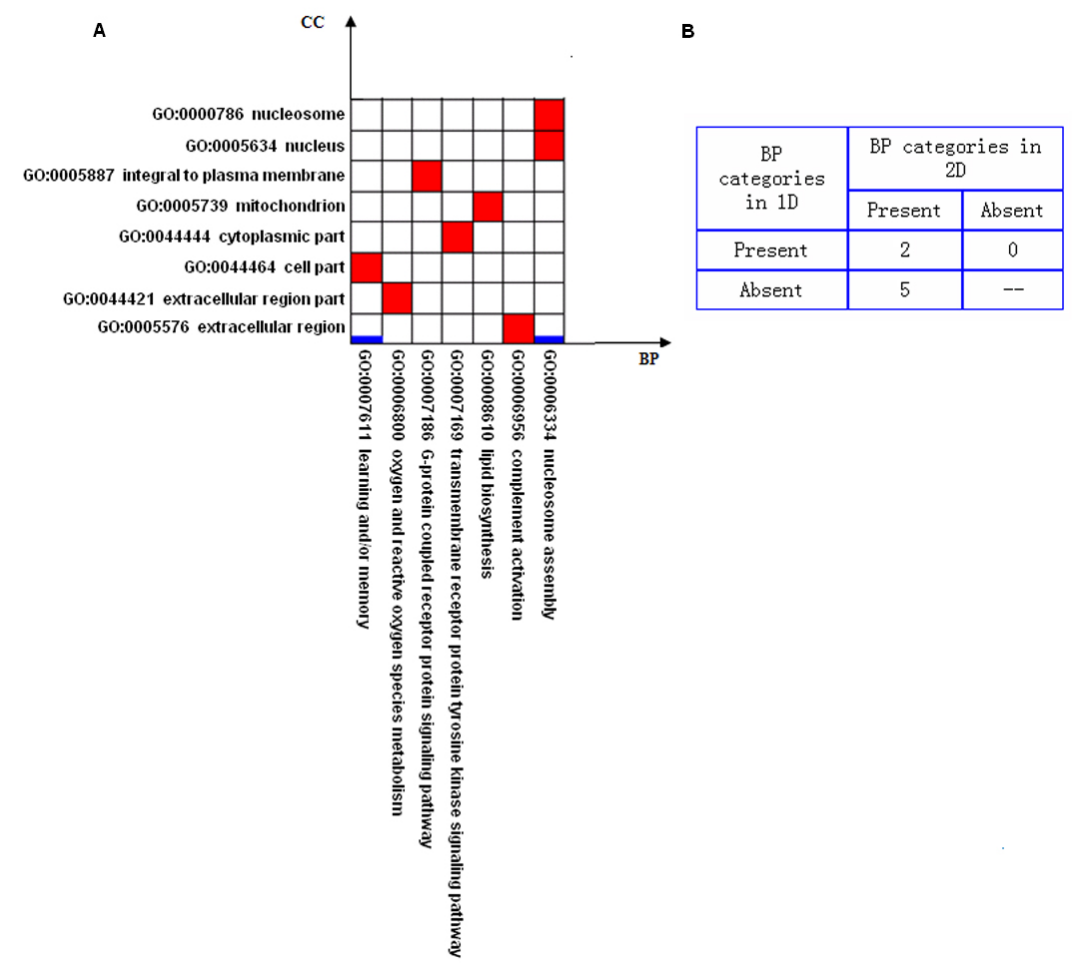
**Comparison between 1-dimensional and 2-dimensional modules in breast cancer**. (A) Horizontal axis represents BP categories ranked by their depths in the BP ontology. Vertical axis represents CC categories ranked by their depths in the CC ontology. The thick blue lines represent the 1-dimensional modules and the red squares represent the 2-dimensional modules. (B) In the confusion matrix, we show the numbers of BP categories which are present in both 1-dimensional and 2-dimensional modules; present in 1-dimensional but absent in 2-dimensional modules; absent in 1-dimensional but present in 2-dimensional modules.

**Figure 5:**
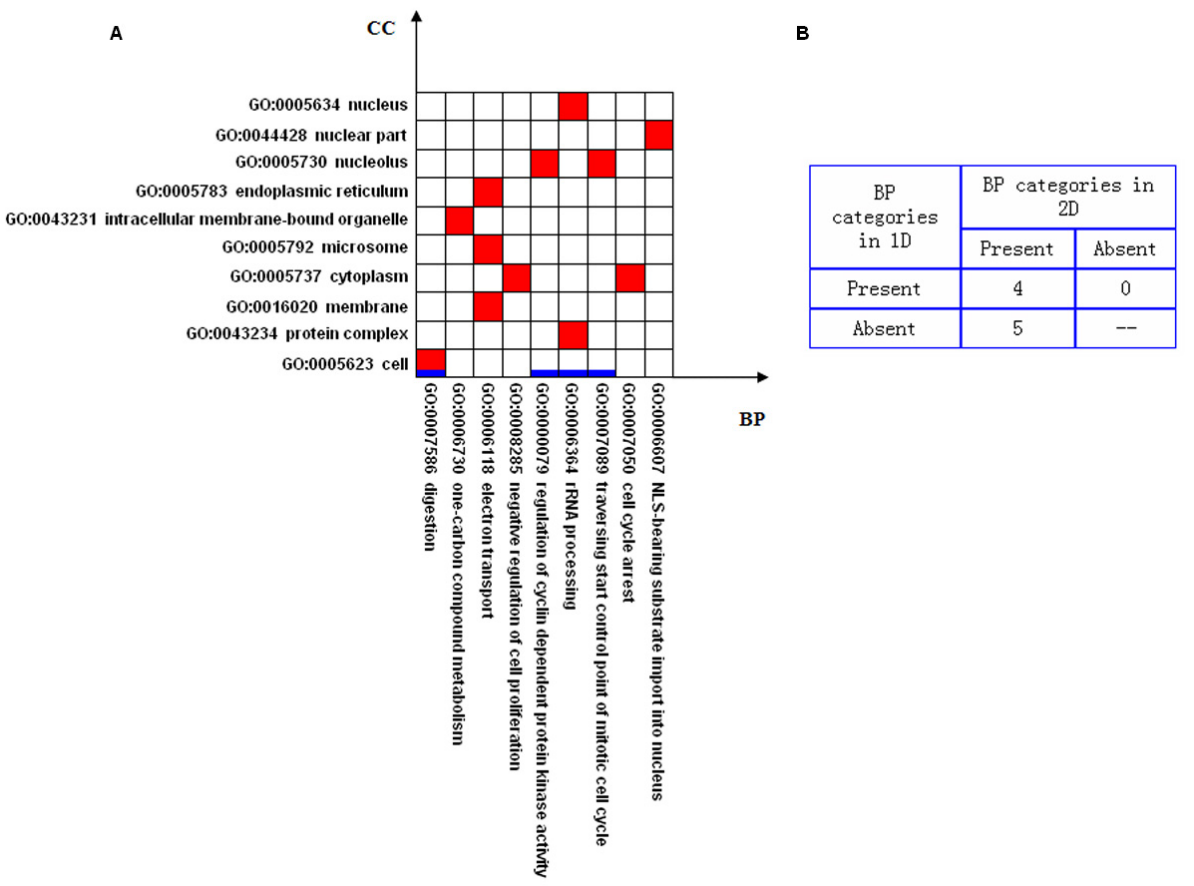
**Comparison between 1-dimensional and 2-dimensional modules in gastric cancer**. (A) Horizontal axis represents BP categories ranked by their depths in the BP ontology. Vertical axis represents CC categories ranked by their depths in the CC ontology. The thick blue lines represent the 1-dimensional modules and the red squares represent the 2-dimensional modules. (B) In the confusion matrix, we show the numbers of BP categories which are present in both 1-dimensional and 2-dimensional modules; present in 1-dimensional but absent in 2-dimensional modules; absent in 1-dimensional but present in 2-dimensional modules.

(8) Redundancy treatment: GO-2D suggests an empirical way to reduce the redundancy among the resulting 2-dimensional modules identified in the hierarchical structure of GO. When some modules share a same primary category in the primary tree (e.g. BP), GO-2D focuses on the combined category containing the most specific secondary category in the secondary tree (e.g. CC).

**Figure 6:**
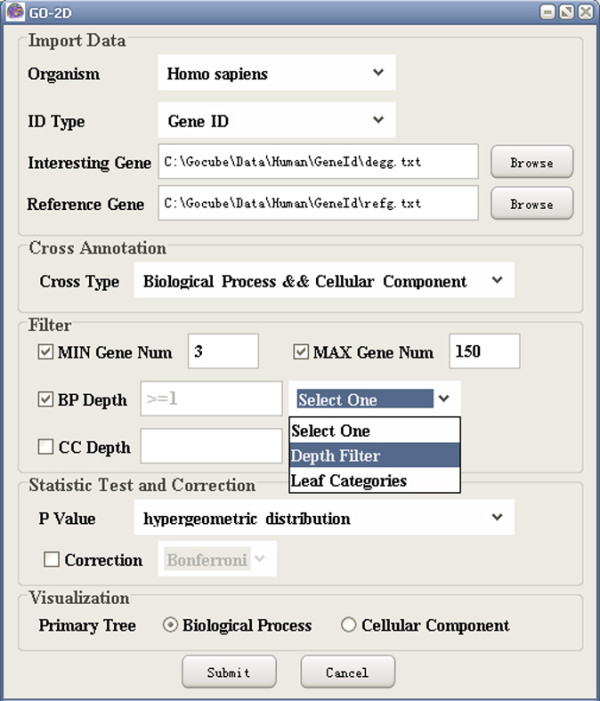
A snapshot of GO-2D: depth selection.

**Figure 7:**
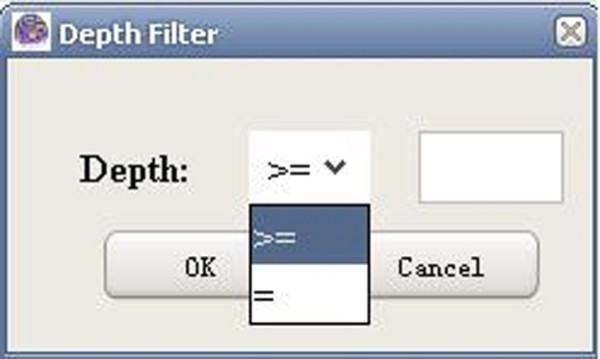
A snapshot of GO-2D: depth filter.

**Figure 8:**
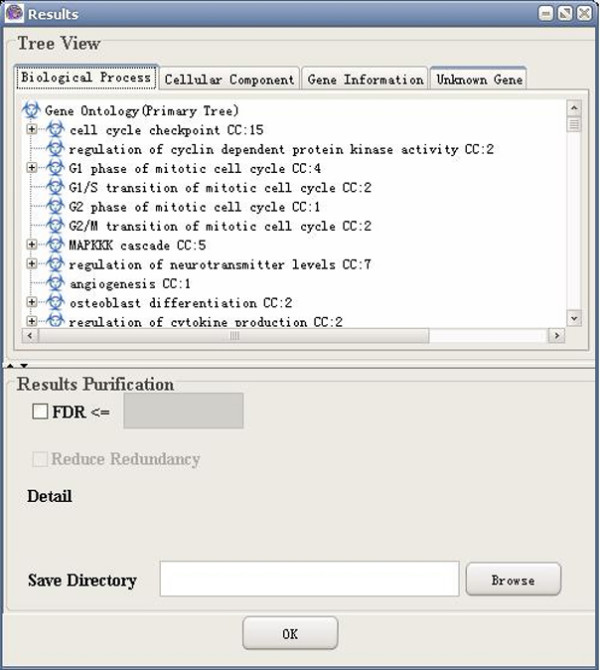
A snapshot of GO-2D: primary tree view.

**Figure 9:**
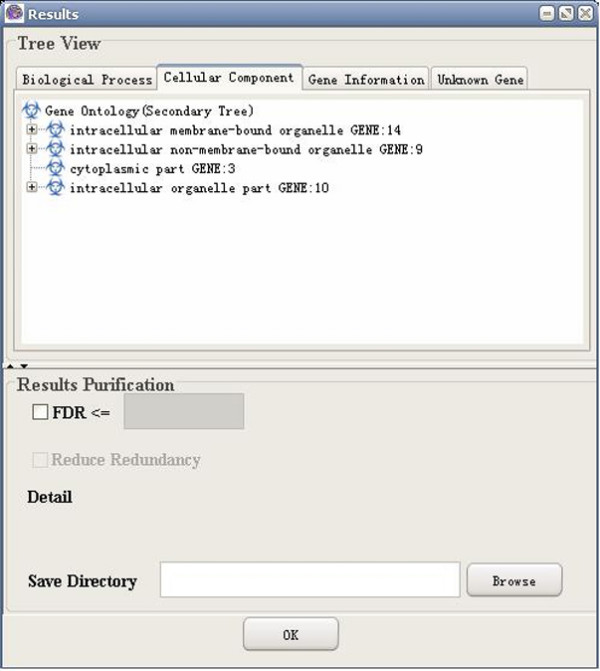
A snapshot of GO-2D: secondary tree view.

**Figure 10:**
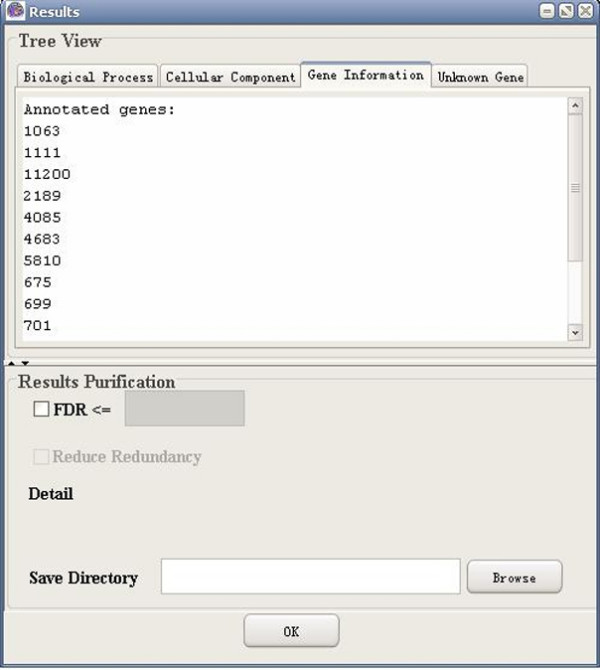
A snapshot of GO-2D: gene information (Entrez gene).

**Figure 11:**
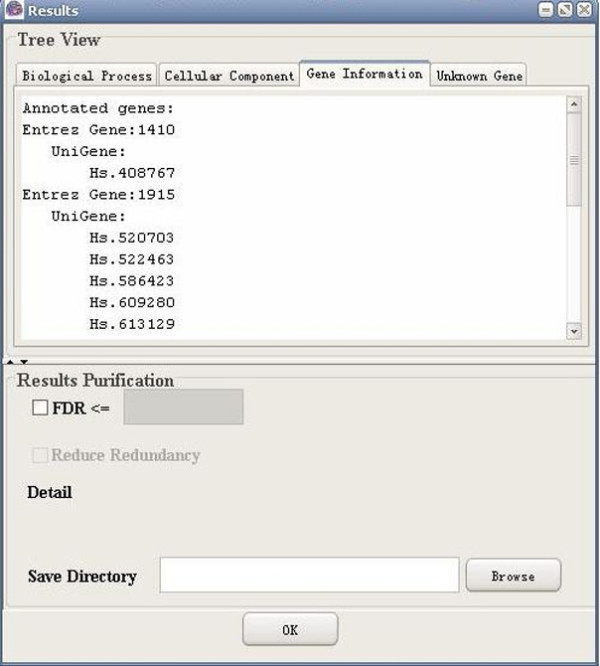
A snapshot of GO-2D: gene information (UniGene).

**Figure 12:**
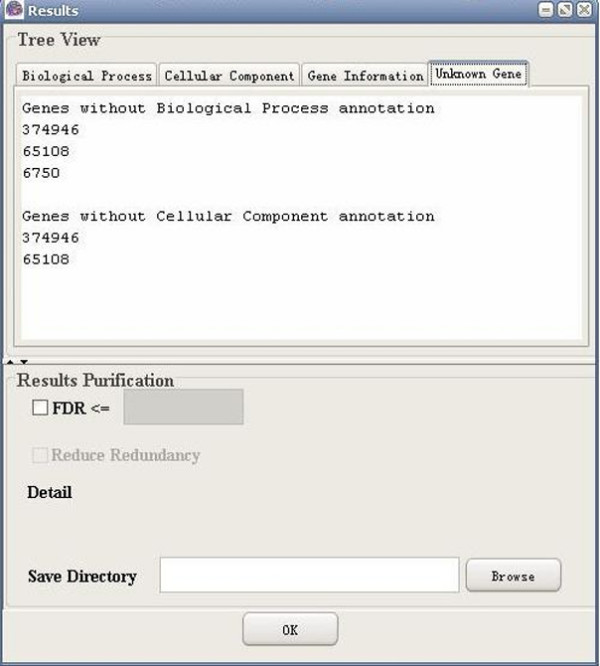
A snapshot of GO-2D: unknown gene (Entrez gene).

**Figure 13:**
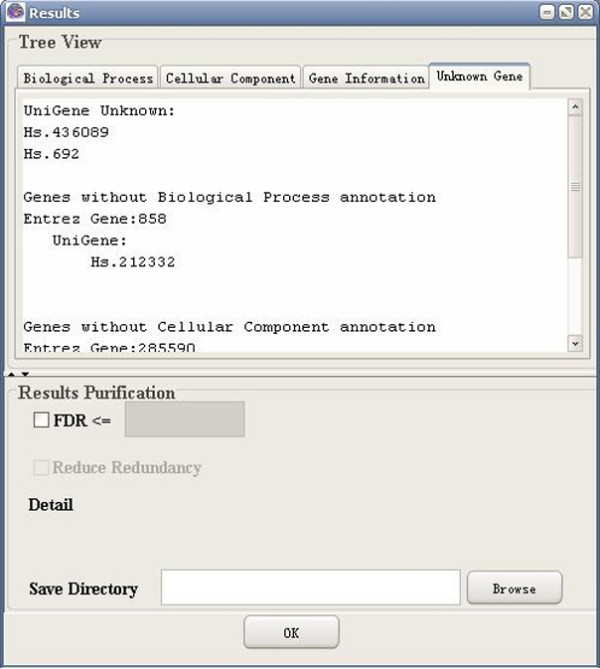
A snapshot of GO-2D: unknown gene (UniGene).

**Figure 14:**
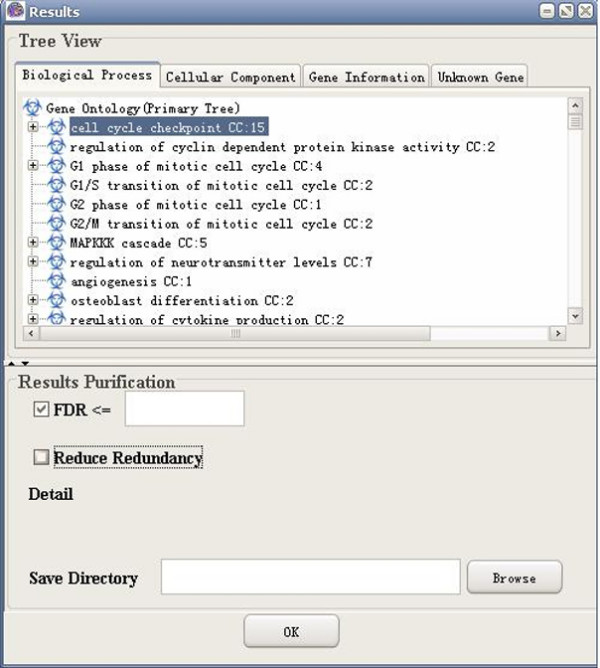
A snapshot of GO-2D: FDR control.

Details are described in the Additional file 1 (Figure 6, 7, 8, 9, 10, 11, 12, 13, 14, 15–Figure 16). Furthermore, GO-2D provides additional standalone software GODAG for visualizing the user selected GO category groups by Directed Acyclic Graph (DAG). In the same DAG, it can show several groups of GO categories marked with different colours, which facilities visual comparisons for the modules identified by different methods (See details in Additional file 2, Figure 17, 18, 19, 20, 21, 22–Figure 23). Also, GO-2D provides another additional standalone tool ConfusionMatrix for comparing the resulting categories identified by 1- and 2-dimensional approaches in GO-2D (see details in Additional file 3, Figure 24, Figure 25).

**Figure 15:**
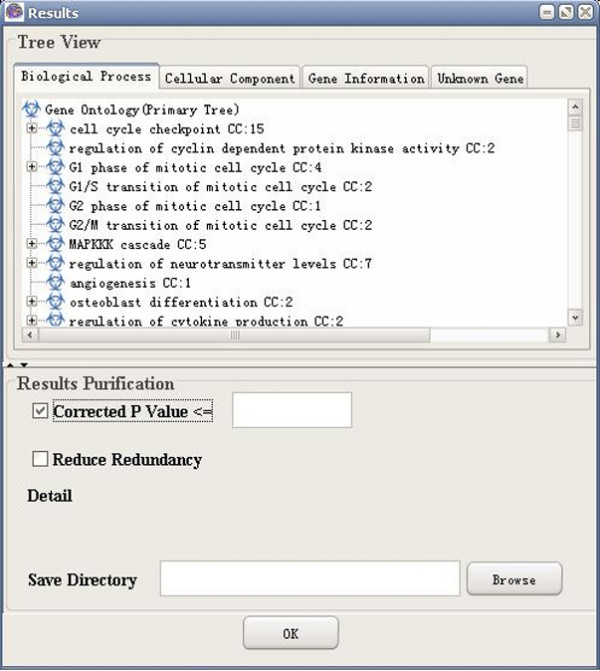
A snapshot of GO-2D: corrected p value (bonferroni).

**Figure 16:**
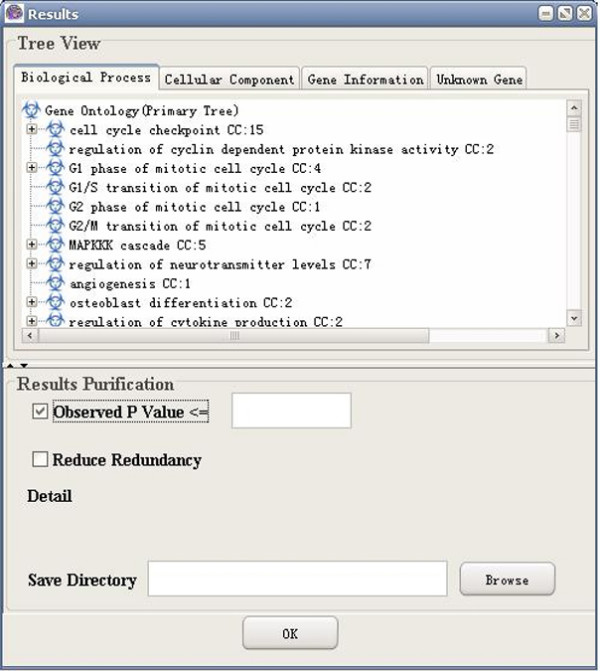
A snapshot of GO-2D: observed p value (no correction is selected).

**Figure 17:**
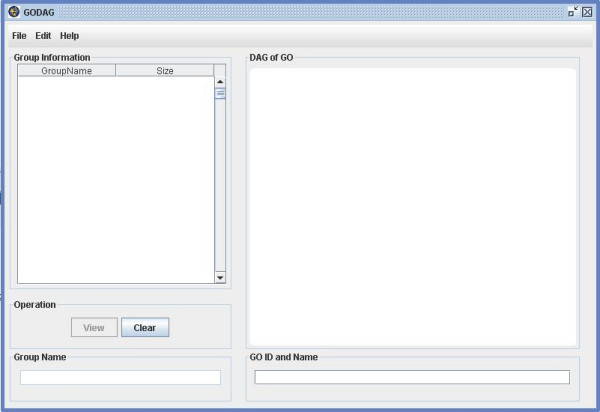
A snapshot of GODAG: the main user interface.

**Figure 18:**
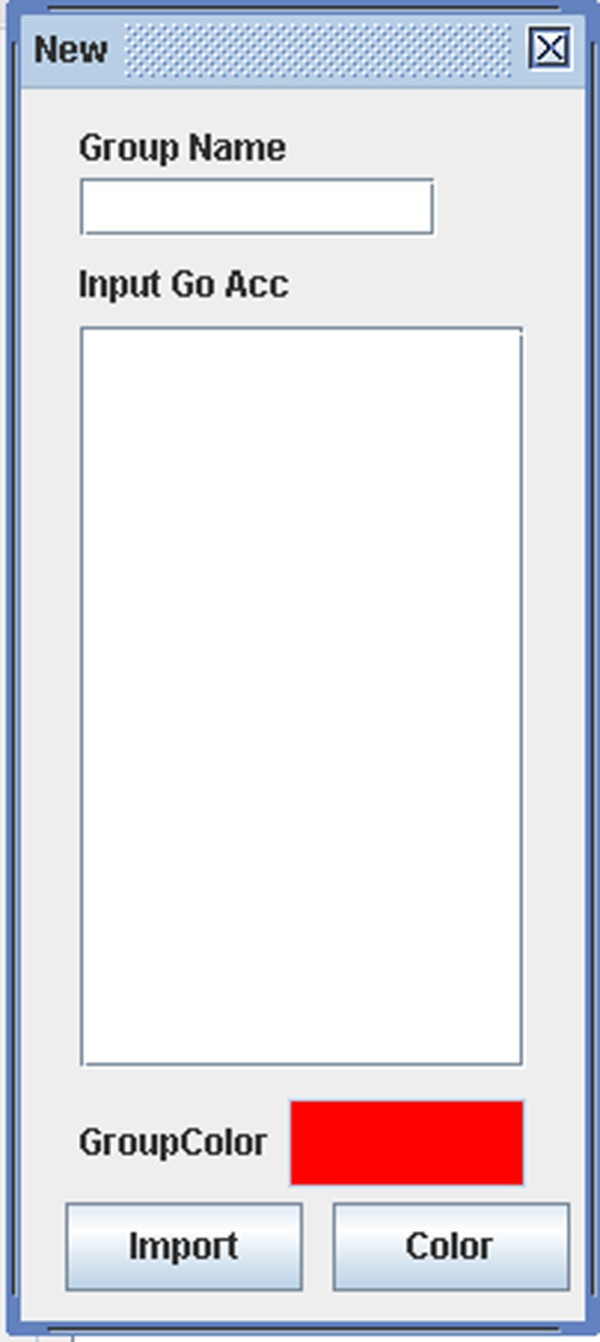
A snapshot of GODAG: input page.

**Figure 19:**
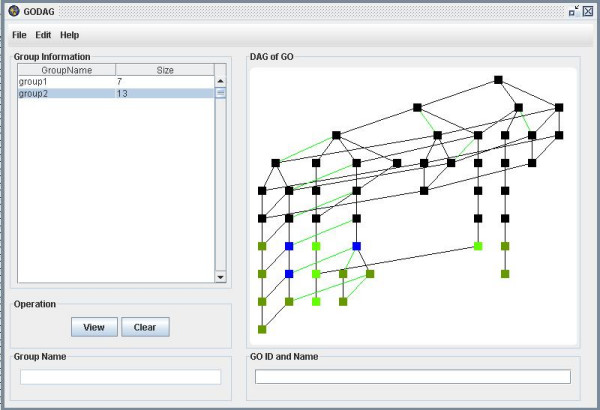
A snapshot of GODAG: result page.

**Figure 20:**
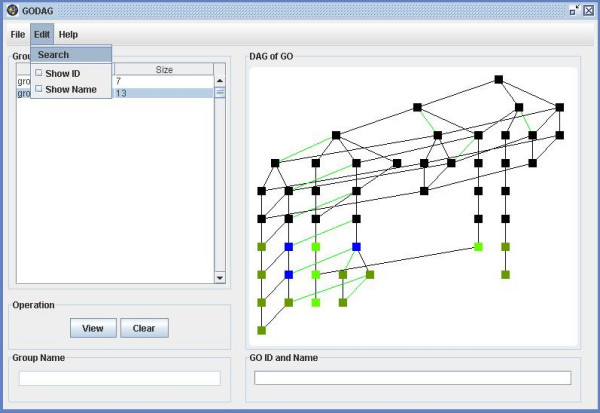
A snapshot of GODAG: edit page.

**Figure 21:**
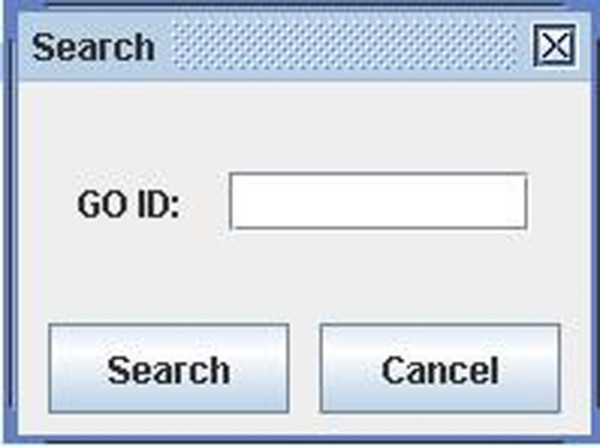
A snapshot of GODAG: search page.

**Figure 22:**
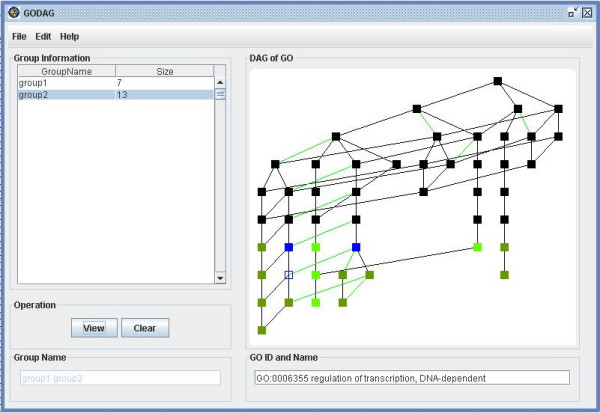
A snapshot of GODAG: the DAG of resulting categories.

**Figure 23:**
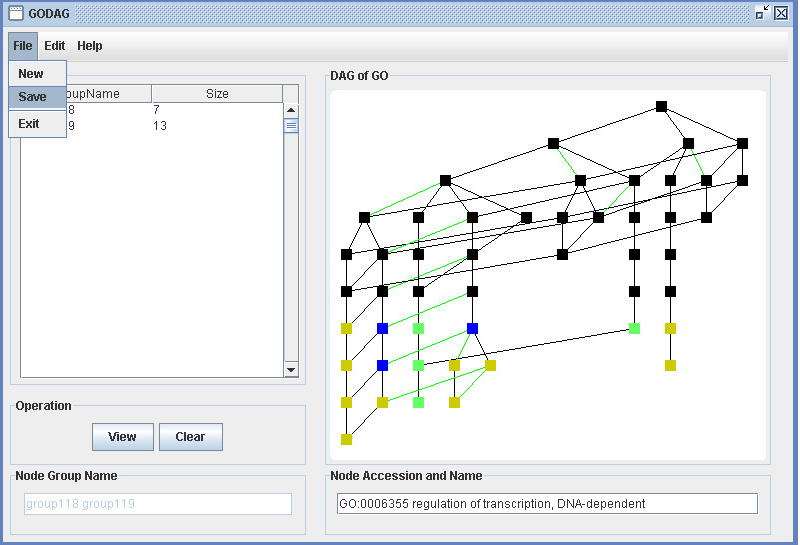
A snapshot of GODAG: save page.

**Figure 24:**
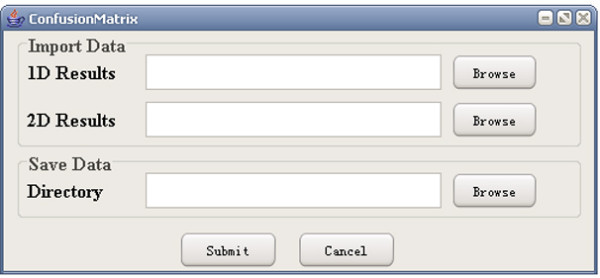
A snapshot of ConfusionMatrix: input page.

**Figure 25:**
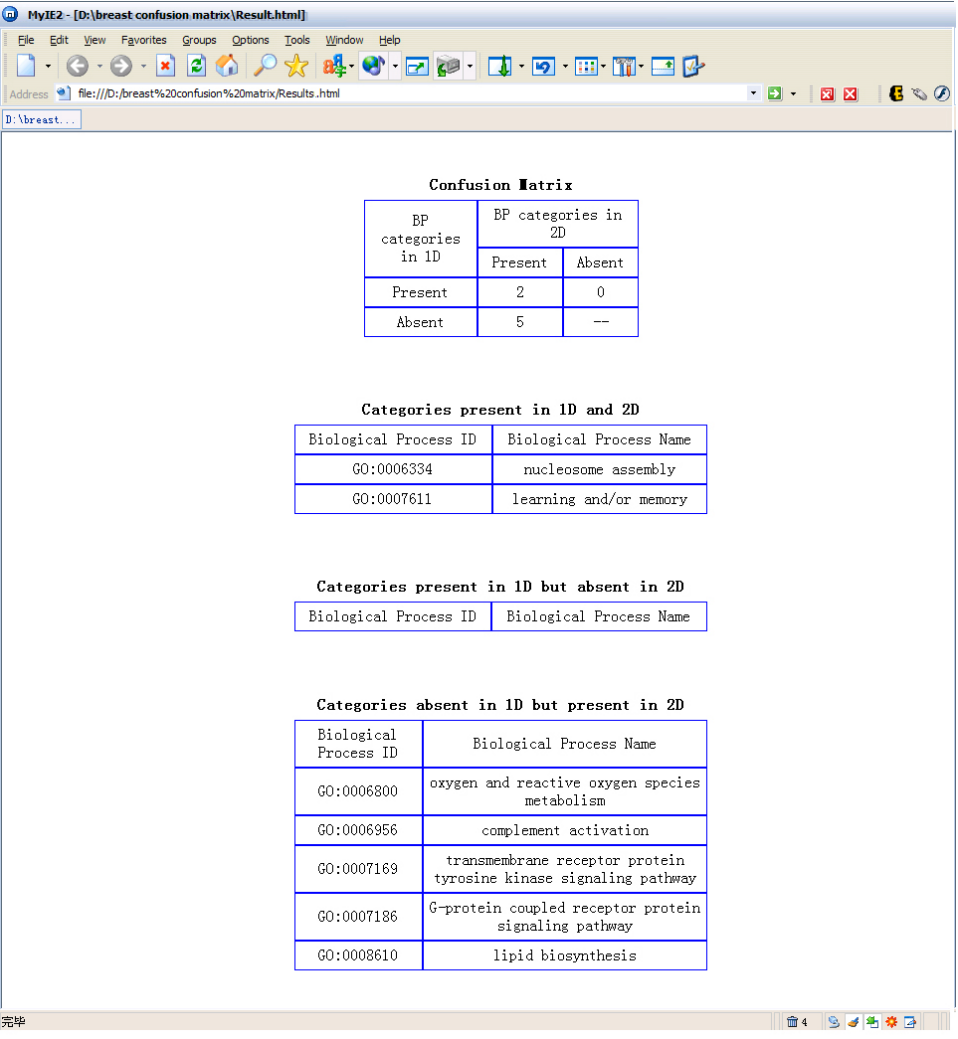
A snapshot of ConfusionMatrix: results page.

### Related software comparison

A recent study [18] has made a detailed comparison of 14 tools for ontological analysis of microarray data. Table 1 compares GO-2D to some typical ones. We highlight that using combined categories for analysis is unique to GO-2D.

**Table 1:** Comparison of GO-2D with related software

	Onto-Express	FatiGO	GoMiner	GOstat	GO-2D
Analysis scope	3 single categories	3 single categories	3 single categories	3 single categories	Combined categories
Correction for multiple tests	Šidák, Holm, Bonferroni, FDR	Step-down minP, FDR [16, 31]	Relative enrichment	Holm, FDR [16]	Bonferroni, FDR [16]
Statistical Analysis	Hypergeometric, Binomial,χ^2^, Fisher's exact test,	Fisher's exact test	Fisher's exact test	χ^2^, Fisher's exact test,	Hypergeometric, Binomial
Visualization	Flat^a^, Tree	Flat^a^, Tree	Tree, DAG	Flat^a^	Tree, DAG
Application	Web	Web	Stand-alone	Web	Stand-alone

Flat^a^: The results are shown without hierarchical structure

## Results

Based on the three GO ontologies (Biological_Process, Cellular_Component and Molecular_Function) separately, similar as other tools, GO-2D can also find 1-dimensional modules enriched with interesting genes. Because of the multiple tests problem, the observed *p *value criterion is not justified for comparison, we thus use the same FDR criterion [16] to compare the powers of the approaches to finding 1- and 2-dimensional modules.

### Datasets

The breast cancer dataset contains 20849 genes measured on 21 invasive lobular carcinoma (ILC) and 38 invasive ductal carcinoma (IDC) samples [19]. The gastric cancer dataset contains 20152 genes measured for 103 gastric tumours and 29 normal gastric specimens [20]. Following the pre-processing protocol proposed by Dudoit et al. [21], we eliminate the genes with missing data in more than 5% arrays, apply a base 2 logarithmic transformation for the remaining expression values, and impute the missing values with zeros. Each experiment is normalized to zero median across the genes. The breast and gastric cancer data finally comprise 8575 and 13037genes (Entrez Gene) respectively, of which 318 and 3388 are differentially expressed genes (DEGs) identified by *t*-test with FDR 1%, calculated by BRB ArrayTools [22].

### Parameters

The parameters are set as following:

(1) Hypergeometric distribution

(2) FDR = 0.1

(3) MIN Gene Num 3; MAX Gene Num 150

(4) BP depth = Leaf Categories

(5) CC depth = 1 (for finding 1-dimensional modules only based on BP), or CC depth>= 1 (for finding 2-dimensional cellular-localized functional modules)

(6) Reduce redundancy

For breast cancer (318 interesting genes and 8575 reference genes), it takes about 9 min and 12 min for 1-dimensional and 2-dimensional analysis respectively, with the same computer (CPU: 2.8 GHz and Memory: 1 GB). For gastric cancer (3388 interesting genes and 13037 reference genes), it takes about 16 min and 22 for 1-dimensional and 2-dimensional analysis, respectively.

### Comparison of modules for breast cancer

With the statistical criterion FDR ≤ 0.1, we find eight cellular-localized modules, and two 1-dimensional modules based on BP only. As shown in Figure 4 and described in Table 2 (the details of genes in each module are shown in Additional file 4), we can find that, in addition to the biological processes appeared in the two 1-dimensional modules, the 2-dimensional approach discovers some new specific processes relevant to disease. For example, the biological process 'lipid biosynthesis' is discovered in the cellular-localized module 'lipid biosynthesis & mitochondrion'. Using cellular location information, we find that there are three DEGs among the 10 measured genes that are annotated in this cellular-localized module, and the observed p-value is 0.005 (FDR = 4.8%). However, when we do not use cellular location information, we find four DEGs among the 108 measured genes that are annotated in 'lipid biosynthesis', and the observed p-value is only 0.57 (FDR = 65.6%). This example clearly demonstrates that finding cellular-localized modules is a useful approach to detecting additional disease relevant modules.

**Table 2:** Functional modules for breast cancer (FDR ≤ 0.1)

Dimension	Biological Process Name (# of genes ^d ^in BP)	Cellular Component Name (# of genes ^d ^in CC)	# of genes ^d ^in 2D module	Observed P Value
1D^b^	nucleosome assembly (31)			8.33E-04
	learning and/or memory (7)	---	---	1.58E-03
2D^c^	nucleosome assembly (31)	nucleosome (21)	20	6.19E-05
	nucleosome assembly (31)	nucleus (1779)	31	8.33E-04
	learning and/or memory (7)	cell part (4794)	5	4.78E-04
	oxygen and reactive oxygen species metabolism (36)	extracellular region part (257)	5	4.78E-04
	lipid biosynthesis (108)	mitochondrion (376)	10	4.99E-03
	complement activation (14)	extracellular region (388)	11	6.68E-03
	transmembrane receptor protein tyrosine kinase signalling pathway (73)	cytoplasmic part (1297)	11	6.68E-03
	G-protein coupled receptor protein signalling pathway (172)	integral to plasma membrane (459)	61	7.07E-03

1D^b^: Functional modules based on biological process (one-dimensional modules).

2D^c^: Cellular-localized functional modules (two-dimensional modules).

# of genes ^d^: the numbers of genes from the gene expression dataset (annotated in the original BP/CC categories or 2-dimensional modules)

A cellular-localized module identified is "BP: oxygen and reactive oxygen species metabolism" in "CC: extracellular region part". Oxidative stress (generating reactive oxygen species) has been linked to cancer initiation and progression. It has been suggested [23] that G. lucidum inhibits the oxidative stress-induced invasive behavior of breast cancer cells by modulating extracellular signal-regulated protein kinases signalling.

For the cellular-localized module "BP: lipid biosynthesis" in "CC: mitochondrial", Zhao, et al. [19] suggested that lipid/fatty acid metabolism may be partially responsible for different proliferation rates of tumor cells in ILCs and IDCs. In addition, mtDNA polymorphisms may be underappreciated factors in breast carcinogenesis [24].

The third example is "BP: G-protein coupled receptor protein signalling pathway" in "CC: integral to plasma membrane", Holland JD et al [25] showed that CXCR4 is subject to controlled regulation in breast cancer cells via differential G protein-receptor complex formation, and this regulation may play a role in the transition from nonmetastatic to malignant tumors.

The last example is for the "BP: complement activation" in "CC: extracellular region". Caragine TA et al. provided direct in vivo evidence that an inhibitor of complement activation can facilitate breast tumor growth by modulating C3 deposition [26].

### Comparison of modules for gastric cancer

With the statistical criterion FDR ≤ 0.1, we find four 1-dimensional modules when based on BP only, and thirteen cellular-localized modules. In addition, as shown in Figure 5 and described in Table 3 (the details of genes in each module are shown in Additional file 4), the 2-dimensional approach detects new disease relevant biological processes combined with the cellular-localization information.

**Table 3:** Functional modules for gastric cancer (FDR ≤ 0.1)

Dimension	Biological Process Name (# of genes ^d ^in BP)	Cellular Component Name (# of genes ^d ^in CC)	# of genes ^d ^in 2D module	Observed P Value
1D^b^	rRNA processing (46)			1.07E-05
	regulation of cyclin dependent protein kinase activity (28)	---	---	2.26E-05
	Digestion (26)			6.04E-04
	traversing start control point of mitotic cell cycle (5)			1.18E-03
2D^c^	rRNA processing (46)	protein complex (1135)	22	2.22E-04
	rRNA processing (46)	nucleolus (73)	20	1.34E-03
	regulation of cyclin dependent protein kinase activity (28)	nucleus (2425)	18	5.27E-05
	Digestion (26)	cell (6889)	16	4.09E-04
	traversing start control point of mitotic cell cycle (5)	nucleus (2425)	5	1.18E-03
	electron transport (229)	endoplasmic reticulum (378)	49	1.53E-04
	electron transport (229)	membrane (2978)	102	2.66E-04
	electron transport (229)	microsome (76)	29	7.80E-04
	cell cycle arrest (50)	cytoplasm (2362)	14	4.77E-04
	negative regulation of cell proliferation (107)	cytoplasm (2362)	37	8.41E-04
	NLS-bearing substrate import into nucleus (12)	nuclear part (465)	5	1.18E-03
	one-carbon compound metabolism (24)	intracellular membrane-bound organelle (3675)	7	1.67E-03

Note: See the footnotes of Table 2.

For example, for the cellular-localized functional module "BP: negative regulation of cell proliferation" in "CC: cytoplasm", Li X et al. [27] suggested that TGF-beta1 affects both proliferation and apoptosis of gastric cancer cells through the regulation of p15 and p21, and induces transient expression of Smad 7 as a negative feedback modulation of TGF-beta1 signal.

Another example is the module "BP: cell cycle arrest" in "CC: cytoplasm". Zheng JY et al. showed that p27 (KIP1) can lead to apoptosis in gastric carcinoma cells [28].

Furthermore, for the same BP, the cellular-localized functional modules are described by additional localization information. For example, for the two cellular-localized modules, "BP: rRNA processing" in "CC: protein complex" and "BP: rRNA processing" in "CC: nucleolus", it has been shown [29] that by reducing the occupancy of the SL1 complex subunits on the rRNA gene promoter and inducing dissociation of the SL1 complex subunits, the transcription of rRNAs is controlled by tumor suppressor PTEN. In addition, a strong correlation has been observed between Nucleolar Organizer regions (loops of DNA encoding ribosomal RNA) counts and metastasis as well as the microscopic type of the gastric carcinoma [30].

## Discussion

When selecting modules from thousands of categories hierarchically structured in GO, the main difficulty is to set statistical significance threshold accounting for the multiplicity of testing. For multiple tests problem, GO-2D adopts the standard methods of Bonferroni correction and FDR control [16,31], which are usually conservative for the non-independent categories organized in ontologies. It has been suggested that re-sampling simulations might be the most reliable way for selecting the significant modules from thousands of categories organized in GO [32]. However, numerical simulations usually suffer from heavy computation burden, and more efficient and feasible re-sampling algorithms deserve further studies [32]. GO-2D outputs the observed p values for the combined categories, which can be used as input data for some more complicated multiple comparisons by existing tools, e.g., the program for Storey's Q value [17].

Since a BP category usually encompasses the genes involved in distinct processes occurring in different cellular compartments [12] and the genes even within a same process may show a clear expression distinction with respect to their cellular localizations [13], the current 1-dimensional approaches are not sufficient enough for identifying the diseases relevant modules. The 2-dimensional approach finds which parts of a BP category, occurring in some cellular compartments, are significantly relevant to disease. As demonstrated by its applications to two cancers in this study, the cellular-localized modules reveal some new biological processes relevant to the diseases in both datasets, in addition to the BPs identified in the 1-dimensional modules. We note that, conceptually, the 2-dimensional approach should cover all BPs identifiable by the 1-dimensional approach, but it might not be always so because of the approximation procedure in the multiple test corrections. Therefore, GO-2D provides both 1- and 2-dimensional approaches for identifying interesting modules of possible disease relevance. When CC Depth is chosen equal to one, the GO-2D just finds only 1-dimensional modules as other software do. Additionally, GO-2D provides the numbers of genes (from the gene expression dataset) annotated in the original BP and CC categories of each 2-dimensional module, so the user can filter the results (e.g., according to the overlapping of the original BP and CC categories) to choose their interesting subsets. We conclude that GO-2D is a useful tool of detecting disease relevant modules for one of the most important routine task of the functional analysis and biological interpretation of the high-throughput microarray data.

In a recent study, we have also shown the power of the 2-dimensional cellular-localized modules for dissecting the heterogeneity of the complex cancers, i.e. discovering disease subtypes by unsupervised clustering analysis [7]. However, there are still open spaces for further improving the module-based analysis approaches. For example, because changes in gene expression patterns can have various forms, different statistical measures (and their thresholds) for finding DEGs and thus the corresponding functional modules should be further explored. Furthermore, we will integrate GO-2D with more data resources in a future version.

## Conclusion

In summary, we have developed a novel tool for identifying the well-characterized 2-dimensional modules, e.g., in terms of both biological processes and cellular locations. The numerical analyses demonstrate that the 2-dimensional functional modules identified in two cancer datasets enjoy explicit relevance to cancer biology, thus suggesting hints for further experiments confirming the novel modular mechanisms.

## Availability and requirements

Project Name: GO-2D

Project home page:

For Windows version: 

For both Windows and Linux version: 

Operating system(s): Windows 2000 (XP) or Linux

Programming language: Java

Other requirements: Java 1.5

License: GNU General Public License

Restrictions to use by non-academics: Contact corresponding author

## Abbreviations

Gene Ontology (GO), biological process (BP), molecular function (MF), cellular component (CC), differentially expressed genes (DEGs).

## Authors' contributions

ZG and JZ described and specified the features of, and problems to be solved by GO-2D; JW implemented coding of the software; MZ, DY, YL, DW and GX participated in testing the program and applied the data mining strategy to the field datasets; all authors participated in reading, approving and revising the manuscript.

## Supplementary Material

Additional File 1 **Manual of GO-2D**. containing the manual of GO-2D, which is a stand-alone tool that identifies 2-dimensional functional modules enriched with interesting genes. Click here for file

Additional File 2 **Manual of GODAG**. containing the manual of GODAG, which is a stand-alone tool that allows the users to visualize their interesting GO categories as a directed acyclic graph. Click here for file

Additional File 3 **Manual of ConfusionMatrix**. containing the manual of ConfusionMatrix, which is a tool for comparing the resulting categories identified by 1- and 2-dimensional approaches in GO-2D. Click here for file

Additional File 4 **Gene information**. spreadsheet containing the names of all the genes in the modules shown in Table 2 and Table 3. Click here for file
