# Supplementary material for: GO-2D: identifying 2-dimensional cellular-localized functional modules in Gene Ontology
Source: BMC Genomics. 2007 Jan 24;8:30. doi: 10.1186/1471-2164-8-30 (PMC1794235; doi:10.1186/1471-2164-8-30)
Supplement: Additional File 1 — Manual of GO-2D. containing the manual of GO-2D, which is a stand-alone tool that identifies 2-dimensional functional modules enriched with interesting genes. [file 1471-2164-8-30-S1.pdf]

Manual of GO-2D

Introduction

Based on the combined categories from Gene Ontology, GO-2D is a stand-alone tool that identifies 2-dimensional functional modules enriched with interesting genes (e.g., differentially expressed genes) from a genome-scale experiment (e.g. DNA microarrays).

GO-2D can be run in MS-Windows and Linux. The user with Linux operating system should do the following preparation before using GO-2D:

1. **Install Java program in Linux**

   To install Java 1.5, the user can run the command "./jdk-1_5_0_02-linux-i586-rpm.bin" in "Terminal".

2. **Configure the Java environment variables**

   (1) Firstly, the user should run the command "vi /etc/profile.d/java.sh" to create the "java.sh" file.

   Then, to configure the Java environment variables, the user should press the "i" key to enter the insert mode and then add the following sentences:

   #set java environment

   JAVA_HOME=/usr/java/jdk1.5.0_02

   CLASSPATH=.:$JAVA_HOME/lib/tools.jar

   PATH=$JAVA_HOME/bin:$PATH

   export JAVA_HOME CLASSPATH PATH

   in the "java.sh".

At last, the user can press the “ESC” key to exit VI Editor insert mode and then input “Ctrl:wq” to save the “java.sh” file and quit the VI Editor.

(2) To check whether the JDK has been successively installed, the user can run the command “java –version” in “Terminal”. If “Terminal” shows the version of JVM, the JDK has been installed successively.

3. Install SQLite database

SQLite can be downloaded from our web site (http://www.hrbmu.edu.cn/go-2d/software.htm). The user should put the SQLite installation file in the “tmp” folder and then implement the following commands in “Terminal” to finish the installation.

(1) cd /tmp

(2) tar –xzvf sqlite-3.3.4.tar.gz

(3) cd sqlite-3.3.4

(4) ./configure --disable-tcl

(5) make

(6) make install

4. Run GO-2D

The user can implement the command “./go-2d.sh” to run GO-2D (**Note:** Since some configurations of the native library should be done in the file “go-2d.sh”, only running the command “java –jar go-2d.jar” does not work.)

Instructions

1. Select the organism

GO-2D can deal with genes from different organisms, including Homo sapiens, Drosophila melanogaster, Caenorhabditis elegans, and Saccharomyces cerevisiae. The organism for analysis can be selected by clicking on the drop down list next to "Organism" (Fig. 1).

2. Select the ID type

The user can select ID type after selecting one organism. GO-2D can be queried using Entrez Gene and UniGene for human and organism specific IDs in GO for the other three species.

3. Import the interesting and reference genes

After selecting the "Organism" and "ID Type", the user can input interesting and reference genes, by clicking the “Browse” button to open up a file dialog box that displays a list of files. After selecting an appropriate input file, the user can click the “Open” button in the file dialog box. The Input file must be a TXT file and contain only the selected ID Type in step 2. Each identifier should be listed on a separate line.

4. Cross annotation

The default cross type is “Biological_Process && Cellular_Component”. Other available cross types are “Biological_Process && Molecular_Function” and “Cellular_Component && Molecular_Function”.

5. Filter

In the check box “MIN Gene Num” (and/or “MAX Gene Num”), the user can set the minimum (and/or maximum) number of the reference genes in the combined categories for controlling the levels of abstraction of the resulting combined

categories. By default, this check box is unselected.

If the user selects the check box “BP Depth” (and/or “CC Depth”), a drop down list will be available (Fig. 6). The default depth is “>=1”, which is shown in the text field next to “BP Depth”. If the user selects the “Depth Filter”, the dialog box will appear (Fig. 7). The user can select “>=” or “=” and input a depth level. If the “Leaf Categories” is selected, GO-2D will preserve only the leaf categories (with no subcategories) as the candidate categories.

6. Statistic tests and multiple tests correction

The user can select the probability distribution by clicking the down arrow of the drop down box. The default distribution is "hypergeometric_distribution".

GO-2D provides the Bonferroni and the FDR control [Benjamini, Y. and Hochberg, Y., 1995, Journal of the Royal Statistical Society. Series B (Methodological), 57, 289-300.] for multiple tests correction, which can be selected from the drop down list after the check box is selected.

7. Visualization

The user needs to choose the primary ontology to draw the primary tree.

8. Submit the request

The user clicks the “Submit” button to send the input to the local database. Then, the following processing page shows the information of annotation and calculation (Fig. 2).

9. Results

The default display of the results is the primary ontology tree view (see Fig. 8)

defined in the input page. In the primary ontology tree view (e.g. BP), "CC: n" indicates that there are n categories in the secondary tree (e.g. CC) combined with the corresponding category in the primary tree.

If the user clicks a category in the primary tree, the categories combined with it will be shown in the secondary tree (Fig. 9). If the user clicks a category in the secondary tree, the genes annotated to the corresponding combined category will be shown (Fig. 10). In the secondary tree view, "GENE: n" indicates the number of genes annotated in a combined category. If the user inputs the UniGene cluster ID in the input page, the UniGene ID and the corresponding Entrez Gene ID will be shown (Fig. 11). The genes with no annotation information are shown in the "Unknown Gene" tab panel. If the user inputs the UniGene Cluster ID, the UniGene cluster IDs that cannot be annotated to the Entrez Gene are also shown (Fig. 12, Fig. 13).

The user can select the check box "FDR" (FDR control) or "Corrected P Value <=" (Bonferroni) or "Observed P Value <=" (no correction is selected) to input the threshold (Fig.14, Fig. 15, Fig. 16), and then, the check box "Reduce Redundancy" is available and the details are shown below the check box (Fig. 3). By default, "Reduce Redundancy" is not selected.

At last, the user can save the results by clicking the "Browse" button to select an appropriate directory from the file dialog box displaying a list of files on the user's computer. The user can open the result files in MS-Excel by indicating that the files are semicolon delimited.

The results contain three TXT files: "Combined Category Information.txt",

“Common Interesting Gene.txt”, and “Common Reference Gene.txt”. The “Combined Category Information.txt” collects the following information of the resulting categories: “Biological Process ID”, “Biological Process Name”, “Biological Process Depth”, “Biological Process Gene Num”, “Cellular Component ID”, “Cellular Component Name”, “Cellular Component Depth”, “Cellular Component Gene Num”, “Common Interesting Gene Num”, “Common Reference Gene Num”, “Observed P Value”, “Corrected P Value”. The results show the “corrected p value” for every observed p value. When a total of n combined categories are tested, for the Bonferroni correction, the corrected p value is pn. For the FDR control, let $p(k)$ denote the k-th smallest p value in a total of n combined categories, then its corrected value = $np(k)/k$. The false discovery rate f_k for hypothesis k is bounded by $np(k)/k \le f_k$. If an FDR of f is required for the entire experiment, all hypotheses that satisfy $p(k) \le fk/n$ are declared as significant. When the "Correction" button is not clicked, GO-2D outputs all the observed p values, which can be used in many other existing tools, e.g. the program for Storey’s Q value (http://faculty.washington.edu/jstorey/qvalue), for more complicated multiple tests correction.
