# Supplementary material for: GO-2D: identifying 2-dimensional cellular-localized functional modules in Gene Ontology
Source: BMC Genomics. 2007 Jan 24;8:30. doi: 10.1186/1471-2164-8-30 (PMC1794235; doi:10.1186/1471-2164-8-30)
Supplement: Additional File 2 — Manual of GODAG. containing the manual of GODAG, which is a stand-alone tool that allows the users to visualize their interesting GO categories as a directed acyclic graph. [file 1471-2164-8-30-S2.pdf]

Manual of GODAG

Introduction

GODAG is a stand-alone tool that allows the users to visualize their interesting GO categories as Directed Acyclic Graph (DAG). In the same DAG, it can visualize several groups of GO categories marked with different colors.

GODAG can be run in MS-Windows and Linux. The user with Linux operating system should do the following preparation before using GODAG:

1. **Install Java program in Linux**

   To install Java 1.5, the user can run the command "./jdk-1_5_0_02-linux-i586-rpm.bin" in "Terminal".

2. **Configure the Java environment variables**

   (1) Firstly, the user should run the command "vi /etc/profile.d/java.sh" to create the "java.sh" file.

   Then, to configure the Java environment variables, the user should press the "i" key to enter the insert mode and then add the following sentences:

   ```
   #set java environment
    JAVA_HOME=/usr/java/jdk1.5.0_02
    CLASSPATH=.:$JAVA_HOME/lib/tools.jar
    PATH=$JAVA_HOME/bin:$PATH
    export JAVA_HOME CLASSPATH PATH
   ```

   in the "java.sh".

   At last, the user can press the "ESC" key to exit VI Editor insert mode and then

input “Ctrl:wq” to save the “java.sh” file and quit the VI Editor.

(2) To check whether the JDK has been successively installed, the user can run the command “java –version” in “Terminal”. If “Terminal” shows the version of JVM, the JDK has been installed successively.

3. Install SQLite database

SQLite can be downloaded from our web site (http://www.hrbmu.edu.cn/go-2d/software.htm). The user should put the SQLite installation file in the “tmp” folder and then implement the following commands in “Terminal” to finish the installation.

(1) cd /tmp

(2) tar –xzvf sqlite-3.3.4.tar.gz

(3) cd sqlite-3.3.4

(4) ./configure --disable-tcl

(5) make

(6) make install

4. Run GODAG

The user can implement the command “./godag.sh” to run GODAG (**Note:** Since some configurations of the native library should be done in the file “godag.sh”, only running the command “java –jar godag.jar” does not work.)

Instructions

Input page

1. The user clicks File→New to enter the input page (Fig. 17).

2. In the Input page (Fig. 18), the user inputs the Group Names and the IDs of interesting GO categories (e.g. “GO: 0016070”). It is required to input the GO IDs of the same ontology (Biological Process, Cellular Component, or Molecular Function) in a single column.
3. The user clicks the Color button to choose colors. The default color is red.
4. The user clicks the Import button to submit the GO IDs.

Results

1. After data importing, the names, and sizes of the input groups are shown.
2. The user clicks the “view” button to draw the DAG that displays all the paths from the input GO categories to the root category (Biological Process or Cellular Component or Molecular Function). The GO categories are represented by rectangles in the DAG. The color ones represent the inputted GO categories while the black ones are the necessary linking categories in the paths. Additionally, the color of the overlapped categories between groups is blue by default.
3. In the DAG, the “Is a” relationship between GO categories is shown in black lines while the “Part of” relationship is shown in green lines (Fig. 19).
4. The user can get the GO ID and name of one category by clicking the category in the DAG window (shown in “GO ID and Name”). Furthermore, the group name that the category belongs to can also be found in “Group name”.

Edit

1. The user can click the Edit→Search menu to search a GO category by GO ID

(Fig. 20, Fig. 21). The resulting category is shown as an unfilled rectangle in the DAG (Fig. 22).

2. GODAG can show the GO IDs or the names of all the categories in the DAG by clicking Edit→Show Accession or Edit→Show Name.

Save

The user can click the File→Save menu to open up a file dialog box that displays a list of files on the user's computer, and then navigate to select an appropriate save path and input a file name (Fig. 23). After inputting the file name, the user can click the "Save" button to save a JPEG file.
