# Supplementary material for: GO-2D: identifying 2-dimensional cellular-localized functional modules in Gene Ontology
Source: BMC Genomics. 2007 Jan 24;8:30. doi: 10.1186/1471-2164-8-30 (PMC1794235; doi:10.1186/1471-2164-8-30)
Supplement: Additional File 3 — Manual of ConfusionMatrix. containing the manual of ConfusionMatrix, which is a tool for comparing the resulting categories identified by 1- and 2-dimensional approaches in GO-2D. [file 1471-2164-8-30-S3.pdf]

Manual of ConfusionMatrix

Introduction

ConfusionMatrix is a tool for comparing the resulting categories identified by 1-dimensional (1D) and 2-dimensional (2D) approaches in GO-2D. It outputs a confusion matrix showing the relationship between those categories.

ConfusionMatrix can be run in MS-Windows and Linux. The user with Linux operating system should do the following preparation before using ConfusionMatrix:

1. **Install Java program in Linux**

   To install Java 1.5, the user can run the command "./jdk-1_5_0_02-linux-i586-rpm.bin" in "Terminal".

2. **Configure the Java environment variables**

   (1) Firstly, the user should run the command "vi /etc/profile.d/java.sh" to create the "java.sh" file.

   Then, to configure the Java environment variables, the user should press the "i" key to enter the insert mode and then add the following sentences:

   ```
   #set java environment
    JAVA_HOME=/usr/java/jdk1.5.0_02
    CLASSPATH=.:$JAVA_HOME/lib/tools.jar
    PATH=$JAVA_HOME/bin:$PATH
    export JAVA_HOME CLASSPATH PATH
   ```

   in the "java.sh".

   At last, the user can press the "ESC" key to exit VI Editor insert mode and then

input “Ctrl:wq” to save the “java.sh” file and quit the VI Editor.

(2) To check whether the JDK has been successively installed, the user can run the command “java –version” in “Terminal”. If “Terminal” shows the version of JVM, the JDK has been installed successively.

3. Run ConfusionMatrix

The user can implement the command “./confusion_matrix.sh” to run ConfusionMatrix (**Note:** Since some configurations of the native library should be done in the file “confusion_matrix.sh”, only running the command “java –jar go-2d.jar” does not work.)

Instructions

1. Import Data

To import the categories found in 1D and 2D approaches, the user can click the “Browse” button to open up a file dialog box that displays a list of files. After selecting an appropriate input file, the user can click the “Open” button in the file dialog box. The input file must be the “Combined Category Information.txt” created by GO-2D (Fig. 24).

2. Save Data

The user can save the results by clicking the “Browse” button to select a suitable directory from the file dialog box displaying a list of files.

3. Submit the input and create confusion matrix

After click the “Submit” button, the results will be saved in the chosen path as html (Fig. 25) and txt.

The results consist of four parts: “Results.html”, “Categories present in 1D and 2D.txt”, “Categories present in 1D but absent in 2D.txt” and “Categories absent in 1D but present in 2D.txt”. The user can open the TXT files in MS-Excel by indicating that the files are semicolon delimited.
